# The Sigma-1 Receptor Antagonist, S1RA, Reduces Stroke Damage, Ameliorates Post-Stroke Neurological Deficits and Suppresses the Overexpression of MMP-9

**DOI:** 10.1007/s12035-017-0697-x

**Published:** 2017-08-05

**Authors:** Pilar Sánchez-Blázquez, Andrea Pozo-Rodrigálvarez, Manuel Merlos, Javier Garzón

**Affiliations:** 1 Neuropharmacology. Instituto Cajal, Consejo Superior de Investigaciones Científicas (CSIC), Doctor Arce, 37, 28002 Madrid, Spain; 2 Drug Discovery and Preclinical Development, Esteve, Scientific Park of Barcelona, Baldiri i Reixac 4-8, 08028 Barcelona, Spain

**Keywords:** Sigma 1 receptor, S1RA, Stroke, Neuroprotective effects, MMP-9, Astrogliosis

## Abstract

The glutamate N-methyl-D-aspartate receptor (NMDAR) plays an essential role in the excitotoxic neural damage that follows ischaemic stroke. Because the sigma-1 receptor (σ1R) can regulate NMDAR transmission, exogenous and putative endogenous regulators of σ1R have been investigated using animal models of ischaemic stroke. As both agonists and antagonists provide some neural protection, the selective involvement of σ1Rs in these effects has been questioned. The availability of S1RA (E-52862/MR309), a highly selective σ1R antagonist, prompted us to explore its therapeutic potential in an animal model of focal cerebral ischaemia. Mice were subjected to right middle cerebral artery occlusion (MCAO), and post-ischaemic infarct volume and neurological deficits were determined across a range of intervals after the stroke-inducing surgery. Intracerebroventricular or intravenous treatment with S1RA significantly reduced the cerebral infarct size and neurological deficits caused by permanent MCAO (pMCAO). Compared with the control/sham-operated mice, the neuroprotective effects of S1RA were observed when delivered up to 5 h prior to surgery and 3 h after ischaemic onset. Interestingly, neither mice with the genetic deletion of σ1R nor wild-type mice that were pre-treated with the σ1R agonist PRE084 showed beneficial effects after S1RA administration with regard to stroke infarction. S1RA-treated mice showed faster behavioural recovery from stroke; this finding complements the significant decreases in matrix metalloproteinase-9 (MMP-9) expression and reactive astrogliosis surrounding the infarcted cortex. Our data indicate that S1RA, via σ1R, holds promising potential for clinical application as a therapeutic agent for ischaemic stroke.

## Introduction

Stroke is one of the leading causes of death worldwide with ischaemic stroke accounting for the clear majority of cases [1, 2]. The global 30-day mortality rate for stroke is estimated at around 23%, but mortality rates can vary according to age, gender and country [3]. Although a variety of rehabilitation strategies exist, no currently approved drug-based therapy can stimulate the recovery of neurological functions in patients following stroke [4, 5]. Local inflammatory changes and neural circuit plasticity that occur post-stroke affect the extent of the brain and behavioural recovery. Experimental data suggest that N-methyl-D-aspartate receptor (NMDAR) stimulation can lead to oxidative stress, which can trigger the upregulation of matrix metalloproteinases (MMPs) early after cerebral ischaemia and reperfusion [6, 7]. MMPs, in particular MMP-9, are important mediators of microvascular blood–brain barrier (BBB) injury and haemorrhagic transformation after ischaemic stroke [8, 9]. In addition, the neurotoxicity of glutamate and other excitatory amino acids might play an important role in the pathogenesis of ischaemic neuronal injury [10–12]. Therefore, drugs that antagonise glutamate NMDAR transmission (e.g. memantine) may reduce the volume of cerebral infarction that results from experimental stroke [13].

Evidence suggests that sigma-1 receptors (σ1Rs) are involved in several neurological and psychiatric conditions [14]. Moreover, they have the potential to modulate NMDAR transmission [15]. Recent work has revealed that the histidine triad nucleotide-binding protein 1 (HINT1)–σ1R complex mediates the enhancement that certain G protein-coupled receptors (GPCRs) promote on glutamate NMDAR function [16]. In this context, σ1R antagonism prevents the HINT1 protein from enabling GPCRs to enhance the function of NMDARs, thereby preventing the further enhancement of this glutamatergic activity [16]. In fact, in neuropathic and inflammatory pain models involving NMDAR activation, antagonists of the σ1R are effective in reducing pain behaviours [17, 18]. More relevantly, σ1R ligands have been shown to enhance neuroplasticity and functional recovery following experimental stroke [19]; however, at the doses used, the agonists and antagonists of σ1Rs produce comparable effects. Moreover, the selectivity of some of these pharmacological tools is far from optimal [17]. As a result, the involvement of σ1Rs in these positive effects is debated.

Based on these findings, we explored the possibility that the selective σ1R antagonist, S1RA (E-52862/MR309) [20], could shed light on the role that this receptor plays in the inflammatory response after experimental stroke. We used an animal model of focal ischaemia induced via middle cerebral artery occlusion (MCAO) to reproduce the most frequent form of stroke in humans [21, 22]. Highly reproducible cortical lesions were obtained by directly ligating the middle cerebral artery (MCA) after temporal craniotomy. This primarily damaged the frontal and parietal cortices, thereby resulting in reliable infarct size across animals but limiting their associated sensory deficits [22]. The aim of this study was to determine whether the central and systemic administration of S1RA had therapeutic effects on cerebral infarct and oedema volumes as well as the ability to ameliorate their associated neurological deficits.

## Materials and Methods

### Animals

Wild-type male CD1 mice and knockout mice with the genetic background of CD1 mice and exhibiting targeted disruption of the *σ1R* gene were used in this study (Envigo, Barcelona, Spain). Mice were housed at a constant temperature (22 ± 1 °C) under a 12/12-h light-dark cycle and were allowed unlimited access to food and water. Animal experiments were performed in accordance with the procedures for the Care and Use of Laboratory Animals of the European Commission guidelines (Directive 2010/63/EU). The Committee on Animal Care at Consejo Superior de Investigaciones Científicas (CSIC) approved all procedures for handling and sacrificing the animals.

### Permanent MCAO and the Determination of Infarct Size

Focal cerebral ischaemia was induced via MCAO, as described previously [23]. Briefly, mice were anesthetised, and a vertical skin incision (0.5 cm) was made between the right eye and ear under a dissection microscope. A small craniotomy was performed over the trunk of the right MCA and above the rhinal fissure. The artery was ligated just before its bifurcation between the frontal and parietal branches with a 9-0 suture. Sham-operated animals were subjected to an identical procedure, except that the MCA was not ligated. The mice were returned to their cages after surgery, kept at room temperature and allowed food and water ad libitum. To determine the infarct size 48 h after MCAO, magnetic resonance imaging (MRI) was performed using a BIOSPEC BMT 47/40 (Bruker, Ettlingen, Germany). We used the dorsal third ventricle as an internal anatomical marker to align, register and compare the collection of images from each mouse. The infarct volume was calculated using ImageJ 1.4 as the percentage of the hemisphere that is infarcted based on the T2-weighted images.

Two days after permanent MCAO (pMCAO), one set of animals were euthanised prior to their brains being removed and seven 1-mm-thick coronal brain slides (Brain Matrix, WPI, UK) were obtained. The sections were stained with 1% 2,3,5-triphenyltetrazolium chloride (TTC; Sigma, Spain). Infarct volumes were calculated by sampling each side of the coronal sections with a digital camera (Nikon Coolpix 990, Tokyo, Japan). The extent of unstained infarct area (expressed in mm^2^) was integrated from the total area as an orthogonal projection.

### Drugs

The newly synthesised σ1R antagonist, S1RA: 4-[2-[[5-methyl-1-(2-naphthalenyl)-1H-pyrazol-3-yl]oxy]ethyl] morpholine), was obtained from Laboratorios Esteve (Barcelona, Spain). BD1047 (#0956), BD1063 (#0883) and PRE084 (#0589) were obtained from Tocris Bioscience (Bristol, UK). Compounds were dissolved in ethanol/Cremophor EL/physiological saline (1:1:18). To facilitate selective and straightforward access to their targets, the compounds were each injected into the lateral ventricles of mice at 4 μL as previously described or via an injection in the tail vein. Groups of 8 to 10 mice received doses of the selected compounds.

### Behavioural Outcomes

Behavioural tests were conducted during the first week after pMCAO in S1RA-treated (3 nmol/m icv, 1 h post-surgery) and untreated mice and outcomes compared with sham-operated mice. The primary screening included body weight and contact-righting reflex measurements. Body temperature of each mouse was measured right before, 1, 3 and 5 h following injection using a digital readout thermocouple (BAT-12 thermometer, Physitemp Instruments, Clifton, NJ, USA) with a resolution of 0.1 °C and accuracy of ±0.1 °C attached to a RET-3 Rodent Sensor. Any incidence of abnormal behaviour, fear, irritability, aggression or vocalisation drives to the exclusion of this particular mouse.

#### Activity Meter

Mice were tested individually; locomotion scores were measured using a multicage activity meter (Accuscan activity analyser-Versamax260 v2.4; Omnitech Electronics, Inc., USA). Beam breaks were recorded for 30 m as a measure of basal locomotor activity. Separate counterclockwise (CCW) and clockwise (CW) rotations were also determined. The first measures were obtained 1 day before surgery and 3 days after surgery (i.e. days 2, 3 and 5). The chamber was cleaned with diluted alcohol (70% *v*/*v*) and dried between trials.

#### Hot Plate Test

Animals were individually placed on a hot plate with the temperature adjusted to 53 ± 0.5 °C. A transparent plastic cylinder (14-cm diameter, 20-cm height) was used to confine a mouse on the heated surface of the plate. Heat exposure continued until the nocifensive reaction (licking) of either hind paw occurred.

#### Acoustic Startle Response

The startle device consisted of a non-restrictive Plexiglas cage that enclosed the sensor’s platform but did not touch it. The device was located in a sound-attenuating chamber constantly illuminated via a 10-W lamp. The chamber was also equipped with a loudspeaker that constantly provided 46 dB[A] of background white noise. Two 28-cm loudspeakers (Cibertec, Madrid, Spain), located 15 cm from both sides of the Plexiglas cage, produced the various acoustic stimuli. These speakers were connected to an audio amplifier (Cibertec), which was connected to a noise generator (2001 Function Generator, Columbus, OH, USA). Mice were tested for their acoustic startle response 5 days after surgery. After an initial 5-m period of accommodation to the 46-dB background noise, 15 startle/unpredictable eliciting stimuli were presented (typically 50-ms bursts of 73–120-dB white noise).

#### Passive Avoidance Task

Acquisition and retention of passive avoidance behaviours were examined 5 days after surgery, using two-compartment box with a partition which embodies a sliding door (Ugo Basile, Rome Italy). In the acquisition trial, each mouse was initially placed in the light compartment, and the door between the two compartments was opened after 10 s. When the mouse entered the dark compartment, the guillotine door automatically closed and an electrical foot shock (0.5 mA, 1 s) was delivered through the floor. The latency time before crossing into the dark chamber was recorded. For the retention trial, each mouse was again placed in the light compartment, and the latency time before crossing into the dark compartment was recorded (up to 5 m).

#### Rotarod

Motor coordination was measured using an accelerated rotarod (Ugo Basile). Before surgery, each animal was trained to use the rotarod via six 5-m sessions at a constant acceleration and with an interval of 20 m between trials. On the following days, the mice were again tested and the time to fall from the rod was measured with a cutoff time established at 5 m.

#### Pole Test

The animals were placed head-up on top of a vertical rough-surfaced pole (diameter, 8 mm; height, 55 cm), and the time to orient downward (*t*-turn) and total time to descend were measured. The fastest performance over the three trials was used; mice that dropped from the pole were considered to take 120 s (default value), the highest value.

### Tissue Fixation and Immunohistochemistry

Mice (*n* = 6 per group) were euthanised at 48 h and 5 or 8 days after the induction of pMCAO. Brains were removed after transcardial perfusion, post-fixed by immersion for 3 h at room temperature and cryopreserved in 30% sucrose in 0.1 M phosphate buffer pH 7.4. Brains were frozen and cut in a cryostat (CM1950; Leica Biosystems) and 40-μm coronal sections were collected. Sections were air-dried and blocked at 4 °C overnight using a solution of 0.25% Triton X-100 and 5% donkey serum in Tris-buffered saline (TBS) followed by incubation with anti-glial fibrillary acidic protein (GFAP; Dako Z0334), anti-collagen IV (Abcam AB6586) and anti-neuronal nitric oxide synthase (nNOS; Thermo 61-7000) for 24 h at 4 °C. The signal was revealed using the avidin-biotin peroxidase complex (ABC) technique. A 1:150 dilution of biotinylated *Lycopersicon esculentum* (tomato) lectin (Sigma-Aldrich L0651) was used to stain some sections to study vessel distribution, and processed with an ABC kit followed by diaminobenzidine-nickel. The pictures were taken using a digital camera (Polaroid DMC IE, Cambridge, MA, USA) coupled to a microscope (Axioplan 2, Zeiss, Jena, Germany).

### Synaptosome Preparation and Western Blots

Membranes from the frontal cortex were prepared as previously described [16], and the separated proteins were transferred onto 0.2-μm polyvinylidene difluoride membranes (Bio-Rad #162-0176, Madrid, Spain). The membranes were probed for 24 h at 6 °C with the selected antibodies diluted in TBS + 0.05% Tween 20 (TTBS) in DecaProbe chambers (PR 150, Hoefer-GE, Barcelona, Spain). The primary antibodies were as follows: anti-MMP-9 (1:1000, Millipore AB13458), PhosphoDetect anti-p38 MAP kinase which specifically recognises the dual-phosphorylated (on residues Thr180 and Tyr182) active form of the enzyme (1:1000, Millipore 506119), Phospho-CaMKII (Thr286) antibody (1:1000, Cell Signalling #12716), anti-nNOS (1:1000) and anti-GFAP (1:1000). Signals were detected using horseradish peroxidase (HRP)-conjugated secondary antibodies (1:10,000 in TTBS). Antibody binding was visualised using Immobilon Western Chemiluminescent HRP substrate (Millipore WBKLS0100), and chemiluminescence recorded using a ChemiImager IS-5500 (Alpha Innotech, CA, USA) equipped with a Peltier-cooled CCD camera that provided a real-time readout of 30 frames per second (−35 °C; high signal-to-noise ratio; dynamic range of up to 3.4 optical density units). Densitometry (average optical density of the pixels within the object area/mm^2^) was performed using Quantity One (Bio-Rad, Madrid, Spain).

### Statistical Analyses

All graphs and statistical analyses were generated and performed using the SigmaPlot/SigmaStat v.13 package (SPSS Science Software, Erkrath, Germany). Significance was defined as *p* < 0.05. Data were analysed using paired *t* test or one-way ANOVA followed by all pairwise Holm-Sidak multiple comparison tests.

## Results

### Determination of Infarct Volume

**Fig. 1:**
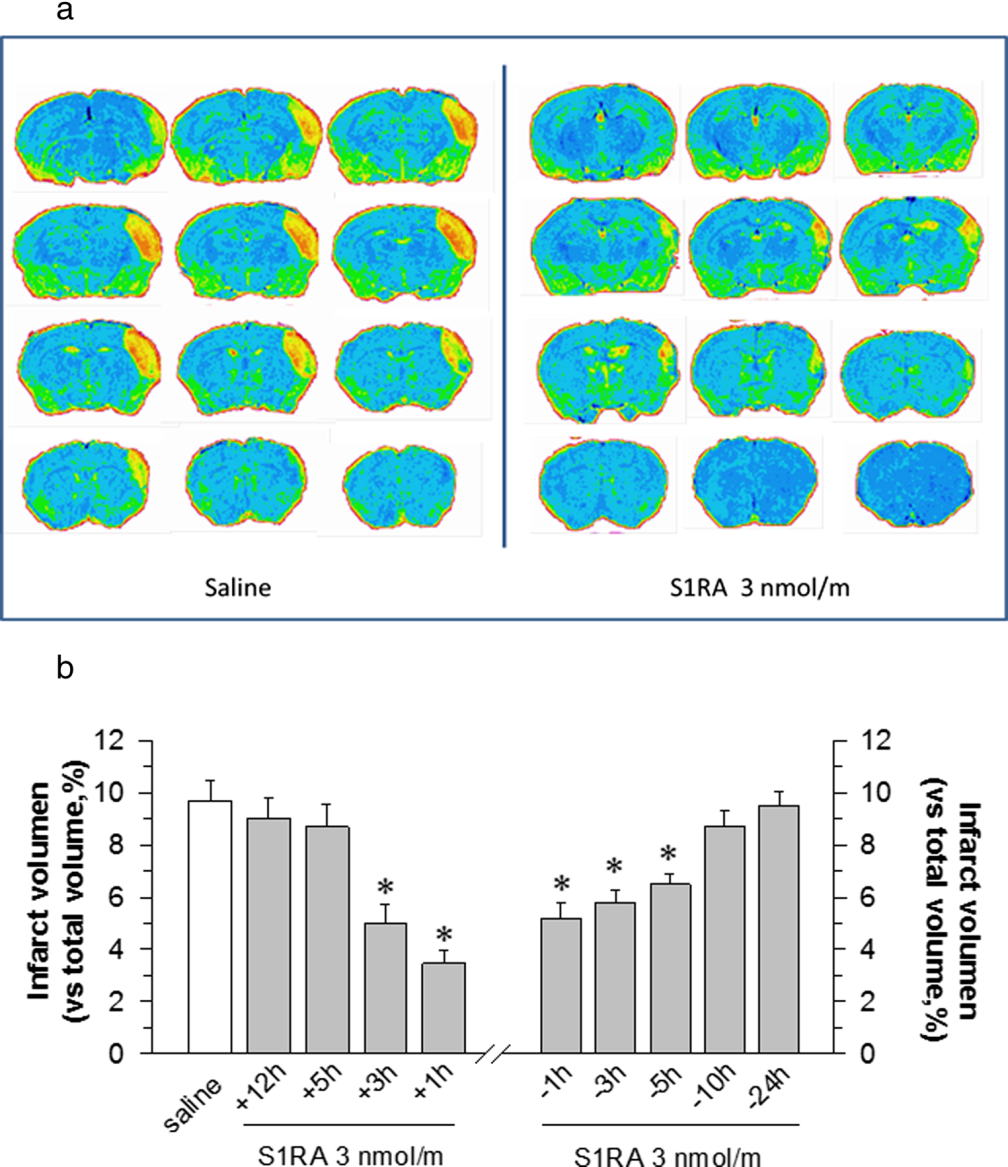
The administration of S1RA diminishes ischaemic brain damage. **a** Representative brain section images were obtained from saline- (*left*) and S1RA-treated mice (3 nmol/mouse, 1 h after surgery; *right*) 48 h after pMCAO using MRI (BIOSPEC BMT 47/40). **b** The bar graphs quantitatively compare the infarct volume (±SEM) from the saline- (*white bars*) and S1RA-treated mice (*grey bars*) at different time intervals before and after surgery. Groups consisted of 8–10 mice, and the data are represented as the means ± SEMs. *Asterisk* indicates the significant difference from saline-treated mice, degrees of freedom (*df*) = 16, all pairwise Holm-Sidak multiple comparison tests following ANOVA, *p* < 0.05

The volumetric analysis of the in vivo MRI data showed that neither surgery nor intracerebroventricular (icv) administration significantly changed the total brain volume (437.9 ± 31.1 and. 456.7 ± 24.8, respectively; sham-operated mice 441.4 ± 26.3 mm^3^). However, pMCAO produced severe injury in mice when examined 48 h after ischaemia (Fig. 1a). Injury was mostly apparent in the cerebral cortex, and the infarct volume was estimated as affecting 9.7 ± 1.8% of the total brain volume. No damage was observed in the sham-operated mice. Compared with untreated mice, the administration of S1RA 1 h post-surgery improved stroke outcomes (an approximate 50% reduction in the infarct size to 3.48 ± 0.9% of the total brain volume) after permanent cerebral ischaemia (Fig. 1a).

To examine the time course of the neuroprotective effect of S1RA, the compound was icv injected at doses of 1, 3 and 10 nmol per mouse. Whereas 1 and 3 nmol showed beneficial effects, the dose of 10 nmol was less effective. Thus, a dose of 3 nmol S1RA was selected for the study, and this dose coincided with that used in animal models of neuropathy and opioid analgesia regulation [16, 24]. Although the protective effect was observed when S1RA was administered up to 5 h before surgery, the beneficial effects disappeared when administered more than 3 h after the initiation of the ischaemic procedure (Fig. 1b).

**Fig. 2:**
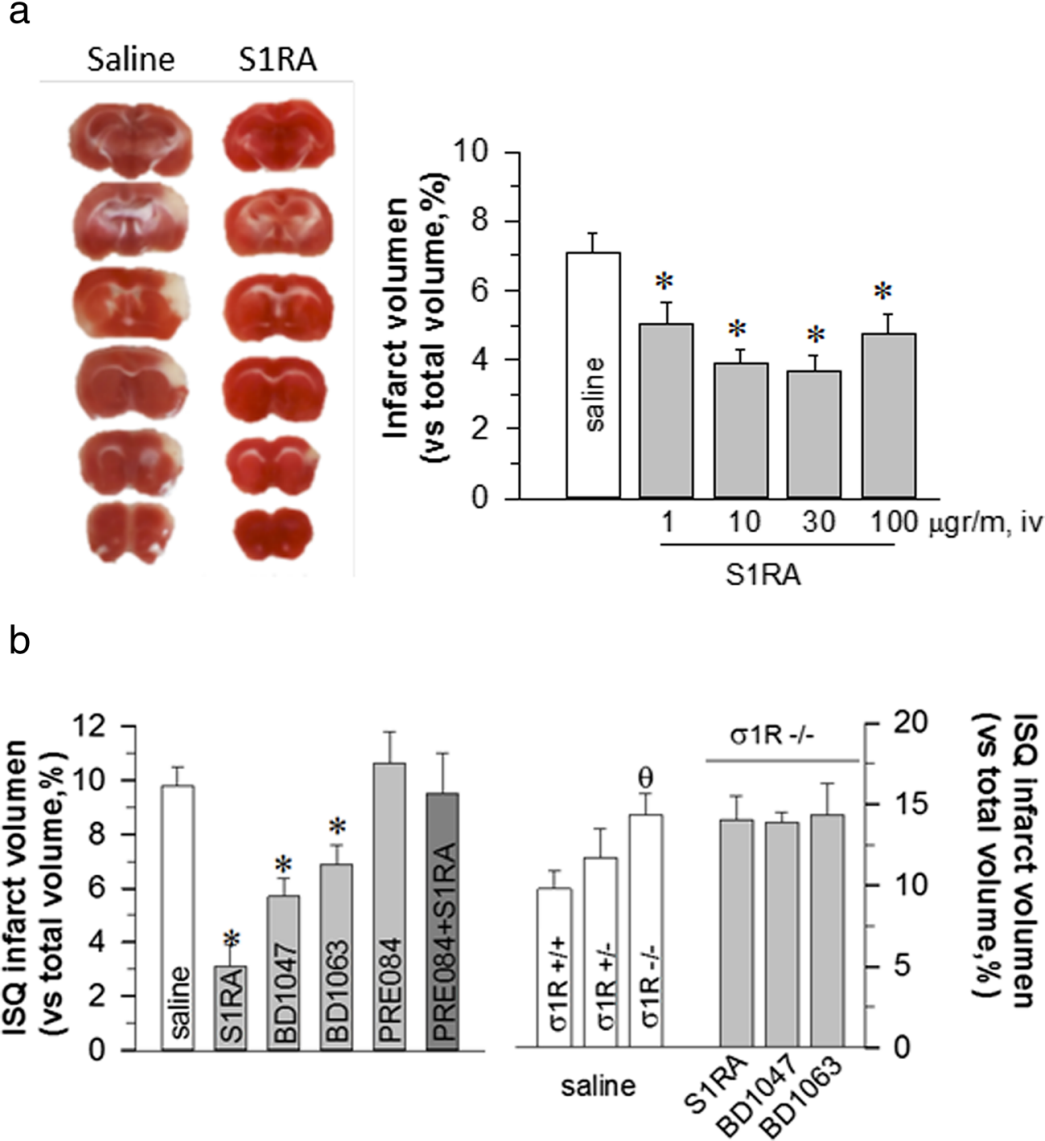
A Left: TTC stained brain section images were obtained from saline and S1RA treated mice (30 mg/mouse, iv; 1 h after surgery) 48 h after pMCAO. White indicates infarction; red staining indicates normal tissue. Right: Quantification of infarct volumes based on TTC staining. B Compounds were injected at a dose of 3 nmol/mouse 1 h after surgery. The *bars* quantitatively compare the infarct volume in WT mice. **σ** 1R^−/−^ animals showed significantly greater infarct areas that were not reduced by S1RA administration. Groups consisted of 8–10 mice, and the data are represented as the means ± SEMs. *Asterisk* indicates the significant difference from saline-treated mice, *θ* significantly different from WT mice; degrees of freedom (*df*) = 16, all pairwise Holm-Sidak multiple comparison tests following ANOVA, *p* < 0.05

Next, we addressed whether the systemic application of S1RA protected against neuronal damage at the end of pMCAO because this protocol has more potential for the clinical treatment of patients recovering from stroke. Intravenous (iv) administration of S1RA 1 h after pMCAO protected against ischaemic injury, showing a similar efficacy as the icv route (Fig. 2a). Compared with untreated mice, S1RA (30 μg per mouse, iv) were clearly protected against stroke outcomes; the infarct sizes were 6.71 ± 1.7 and 3.37 ± 0.9% of the total brain volume, respectively.

We previously reported that S1RA is likely the most potent and selective σ1R antagonist available [24] as other antagonists (e.g. BD1047 and BD1063) that have been used at 3 nmol per mouse produced weaker protective effects. Based on the experimental conditions and doses we used, the PRE084 agonist showed no direct effect on infarct volume; however, it did prevent S1RA neuroprotection (Fig. 2b). The involvement of the σ1Rs in the effects of S1RA was ascertained using σ1R^−/−^ mice. These mice showed increased infarct volumes with respect to their wild-type (WT) controls, which were refractory to the σ1R ligands included in this study (Fig. 2b).

### Behavioural Assessment

**Fig. 3:**
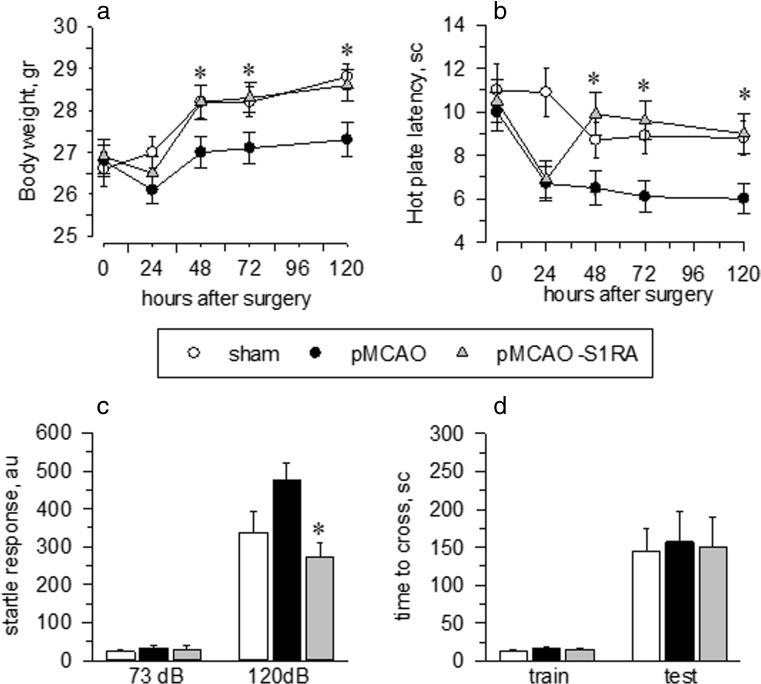
Behavioural recovery from stroke via S1RA treatment. The response rate functions of the three groups of mice regarding **a** body weight gain in grams (g), **b** hot plate latency in seconds (s), **c** startle response at 73 and 120 dB and expressed as arbitrary units (au) and **d** learning performance in the passive avoidance test. Groups of 8–10 mice were used for each treatment, and the data are represented as the means ± SEM. *Asterisk* indicates the significant difference from the sham-operated mice, degrees of freedom (*df*) = 16, all pairwise Holm-Sidak multiple comparison tests following ANOVA, *p* < 0.05

**Fig. 4:**
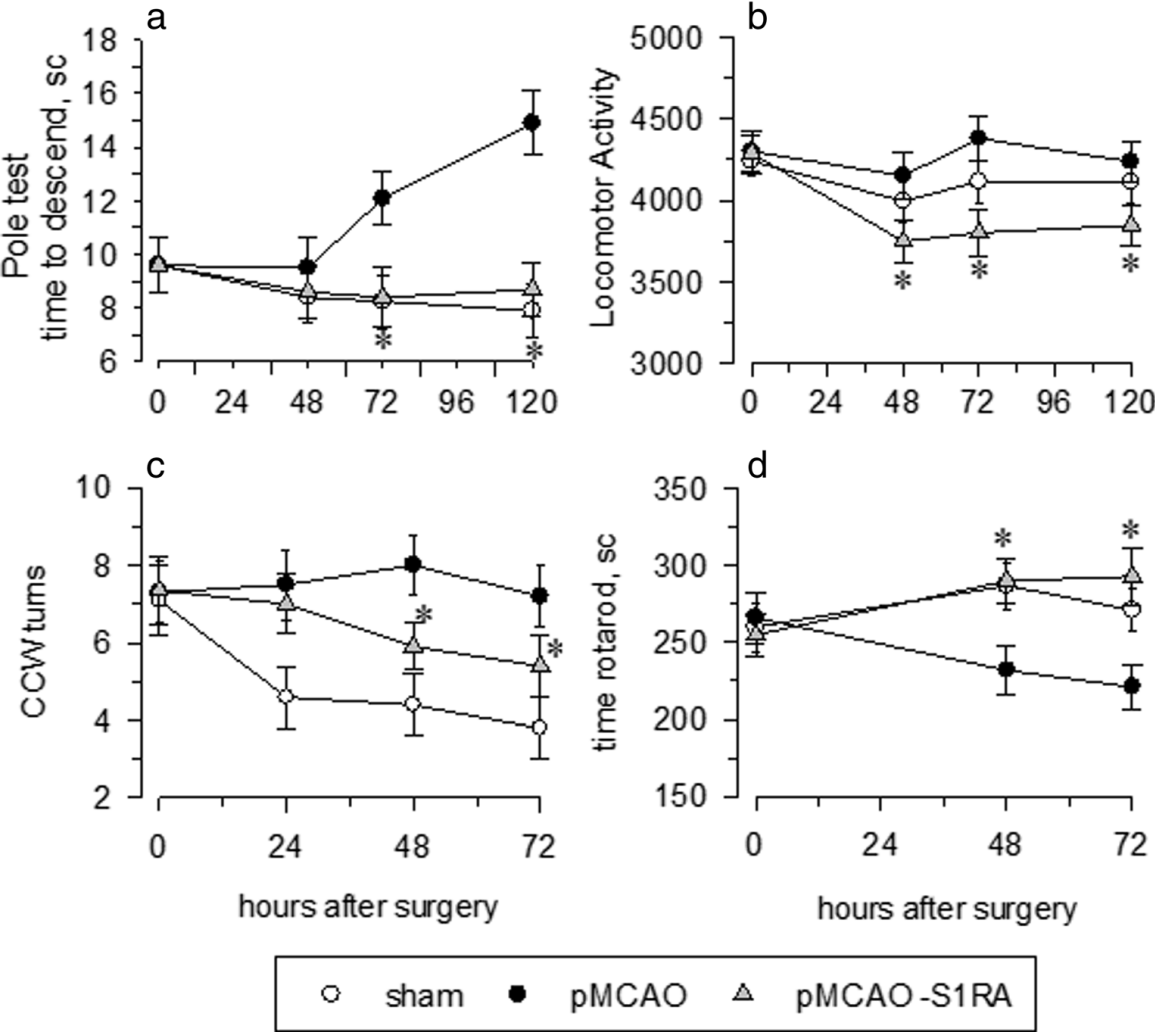
Improved recovery of sensory-motor function from stroke after S1RA treatment. The response rate functions of the three groups of mice regarding the **a** seconds (s) to descent to the floor in the pole test, **b** locomotor activity, **c** counterclockwise (CCW) rotation preference and **d** latency in seconds (s) to fall in rotarod. Groups of 8–10 mice were used for each treatment, and the data are represented as the means ± SEM. *Asterisk* denotes the significant difference from the sham-operated mice, degrees of freedom (*df*) = 16, all pairwise Holm-Sidak multiple comparison tests following ANOVA, *p* < 0.05

In addition to reducing infarct size, we also sought to determine whether S1RA protects or improves the performance of mice in various sensory-motor tests. No differences were observed between the stroke and sham-operated animals with regard to muscle strength or movement latency. Neither alteration in the body temperature of subjects injected with S1RA was observed when compared to saline-injected controls. Rectal temperature was measure 30 min after surgery and then hourly up to 7 h. However, a significant effect of pMCAO was found with regard to body weight (Fig. 3a). Data analyses revealed that the stroke animals weighed significantly less than the sham-operated animals on post-surgical days 2 through 5. To discard the influence of body weight in the behavioural performance, a parallel group of mice with limited access to food were used. Our data indicate that body weight difference does not affect the global behavioural outcomes. Subsequently, we evaluate the influence of pMCAO to heat and acoustic stimuli. In operated animals, an increased sensitivity to both stimulus was observed (Fig. 3b, c). The administration of S1RA (3 nmol/mouse, icv, 1 h after surgery) attenuated all these altered behaviours. No significant difference was observed between the pMCAO and the sham groups with regard to the learning performance task. The latency to enter the dark, electrified chamber was significantly longer on the test day than the training day (5 days post-stroke). However, the majority of the animals entered the dark chamber before the end of the 5-min period, suggesting that retention of this learning task is similar regardless of ischaemic damage (Fig. 3d). According to the vertical-pole task, pMCAO significantly increased the time needed to climb down to the base of the pole (Fig. 4a). The mice developed a hyperactive phenotype, as has been previously reported [25], with a rotation preference and apparent motor deficits (Fig. 4b–d), and S1RA administration significantly attenuated all these behaviours.

### The Effects of S1RA on Post-Ischaemic BBB Disruption, the Inflammatory Response and Astrogliosis

**Fig. 5:**
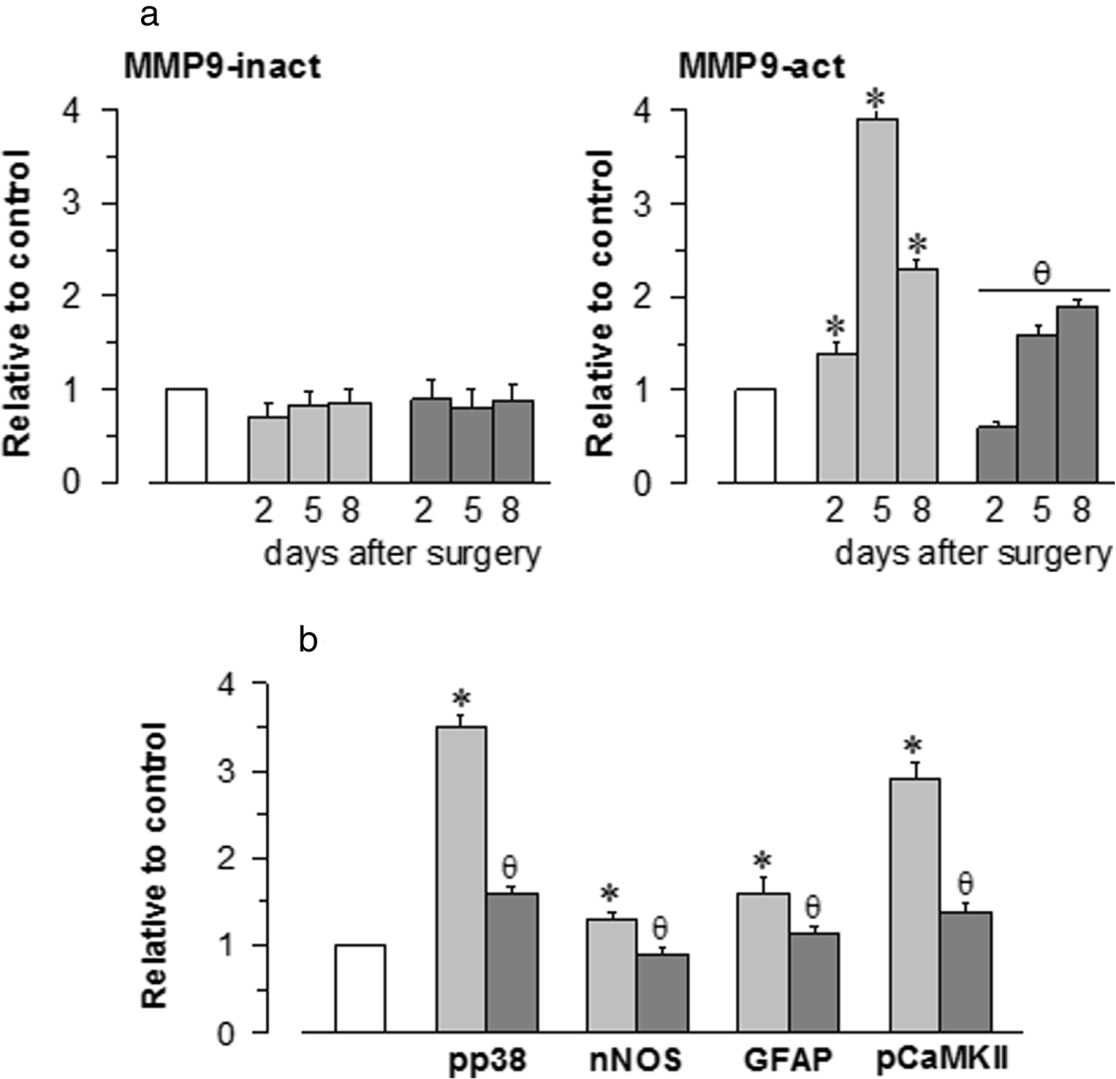
Stimulation of MMP-9 protein expression and inflammatory markers via pMCAO. **a** The expression levels of both the active (86 kDa) and the inactive (92 kDa) forms of MMP-9 were determined at different times after surgery. **b** The ischaemic-induced phosphorylation of p38 and CaMKII as well as the expression levels of nNOS and GFAP were evaluated 8 days after surgery. The enhancing effects of pMCAO (*light grey*) were reduced by S1RA administration (*dark grey*). Each *bar* represents the means ± SEM of the data from three determinations performed using different gels and blots. Groups of 8–10 mice were used for each interval. Immunosignals (average optical density of the pixels within the object area/mm^2^; Quantity One Software, Bio-Rad, Madrid, Spain) were expressed as the change relative to the sham-operated group (attributed an arbitrary value of 1; *white bars*). *Asterisk* denotes the significant difference from the sham-operated mice, *θ* from the pMCAO group, all pairwise Holm-Sidak multiple comparison test following ANOVA, *p* < 0.05

**Fig. 6:**
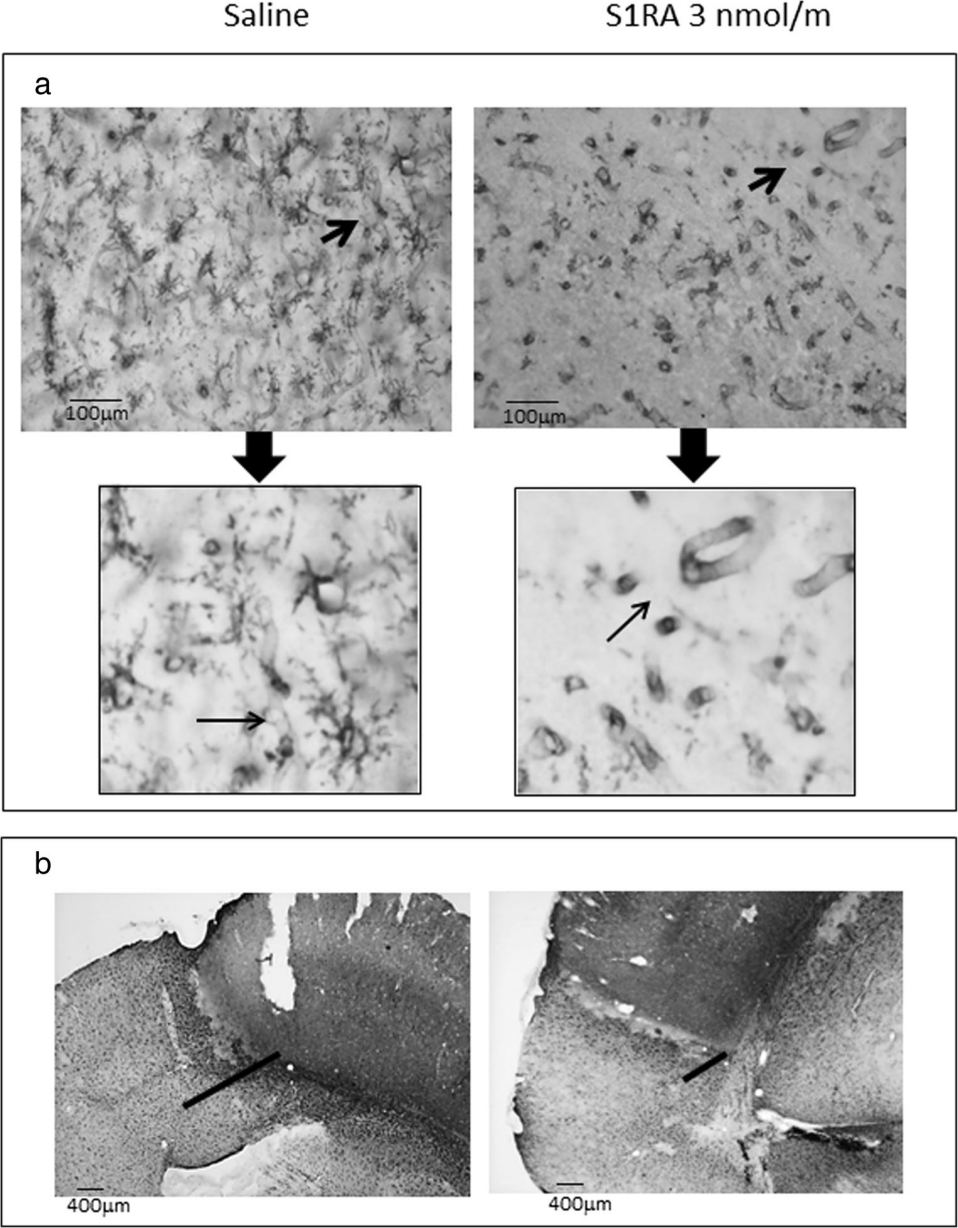
Effect of S1RA administration on cerebral markers changes after pMCAO. Photomicrographs of the mouse ipsilateral cortex showing changes in morphology 48 h after pMCAO. **a** *Lycopersicon esculentum* (tomato) lectin-labelled blood vessels and microglial cells (indicated by the *arrows*). *Insert* amplified area. **b** Glial scar surrounding infarcted zone marked with GFAP. Peri-infarct cortical area of brain from the pMCAO + S1RA group showing significant decrease in microglia expression, and a reduction in the glial scar compared with the pMCAO group. *Scale bars* 100 μm (**a**) and 400 μm (**b**)

Metalloproteinases are associated with cerebrovascular disruption and neuronal damage, and thus, the effects of pMCAO on the MMP-9 protein were determined in western blots. We found that pMCAO produced a significant increase in the activated 86 kDa MMP-9 in the frontal cortex without altering the inactive 92-kDa form (Fig. 5a). These changes were evident 48 h after surgery and they persisted for up to 9 days after stroke. However, the administration of S1RA (3 nmol/m, icv) 1 h after surgery virtually abolished the overexpression of active MMP-9. We also examined p38 MAPK activation as a marker of inflammation, nNOS expression and the endogenous levels of Thr286 phosphorylated CaMKII, indicative of NMDAR over-activation. In addition, glial inflammatory changes were also evaluated in the brain following ischaemic insult, as was the influence of S1RA administration on the reactive astrogliosis induced by pMCAO. Finally, microglial activation was determined through the tomato lectin staining of vessels and microglia, and as expected, pMCAO induced strong reactive astrogliosis as witnessed by GFAP upregulation (Fig. 5b). Immunohistochemical staining of the mouse cortex 48 h after pMCAO showed increased immunoreactivity in astrocytes (GFAP-positive cells) and activated microglia (*L. esculentum* lectin-positive cells; Fig. 6). Significantly, treatment with S1RA diminished the GFAP expression and dampened the activated microglia, as well as the immunoreactivity for nNOS, phosphorylated p38 and pCaMKII (Figs. 5 and 6).

## Discussion

In this study, we used a permanent model of ischaemia to simulate the most common type of localised stroke in humans, specifically the permanent occlusion of the cerebral arteries [26, 27]. We found that the administration of S1RA, a selective antagonist of σ1R, markedly reduced the infarct volume and improved functional recovery. Vehicle-treated animals showed significant post-stroke impairments in motor and sensitive function status, whereas S1RA, given either 1 h after the onset of pMCAO, reduced these deficiencies.

Compounds that are selective agonists or antagonists of σ1R have been used to ascertain whether it is the activation or inhibition of this receptor that results in protection against oxidative stress. Overall, the available in vitro data indicate that inhibition, not activation, of σ1Rs prevents oxidative stress-induced cell death [28]. In that sense, neurosteroids are well known to bind to σ1Rs in addition to hormonal receptors [29]. The neurosteroid progesterone has been considered an antagonist of σ1Rs and has exhibited neuroprotective effects in a number of studies using experimental stroke and CNS trauma models [30–32]. Similarly, the acute administration of the dopamine D2 receptor antagonist haloperidol, at doses that bind to σ1R as an antagonist, also displays neuroprotection [28]. Unfortunately, the available data do not allow us to ascribe the neuroprotective effects of these substances to σ1Rs alone. Importantly, various experimental models of acute brain injury or neurodegeneration have also shown the neuroprotective actions of the σ1R agonists. Rats subjected to 2 h of transient MCAO (tMCAO), continuously treated with the agonist (+) pentazocine or 4-phenyl-1-(4-phenylbutyl) piperidine (PPBB) for 22 h starting 1 h after occlusion [33, 34], showed a reduced infarct volume. A reduction in the infarct size was also found in rats subjected to tMCAO and treated with the PRE084 agonist at 3 h into reperfusion, [35]. Furthermore, dimemorfan has shown protective effects against ischaemic stroke in rats. It has been postulated that these effects are a result of modulation of σ1R-dependent signals, which prevent subsequent glutamate accumulation and the downstream pathologic events [36].

It is unclear whether these ligands act as agonists or antagonists or even whether they interact with σ1Rs to promote neuroprotection. The protective effects of certain σ1R ligands might be because of their effects on other targets such as directly blocking NMDARs. In addition, some data suggest that cutamesine (SA4503), a possible agonist, enhances functional recovery after experimental stroke in rats without affecting infarct size [37]. In a phase 2 clinical trial exploring the safety and functional effects of cutamesine in patients with ischaemic stroke, a trend toward better functional performance with cutamesine compared with placebo was observed, although there was no significant treatment differences in the primary efficacy measures [38].

In the present work, the compounds assayed were administered at equimolar concentrations via the icv route to discard the differences in bioavailability by crossing the BBB. The effects of S1RA were more potent than those of the other evaluated σ1R antagonists. At this dose, PRE084 behaved as an agonist and blocked the positive effects of S1RA and the other σ1R antagonists on infarct volume and functional recovery. This finding corroborates previous data showing that S1RA was the most effective antagonist in an in vivo experimental model that discriminated the agonist/antagonist performance of σ1R ligands [24].

It is interesting to mention that σ1R^−/−^ mice do not reproduce the beneficial effects observed in wild-type mice treated with S1RA. Probably, this difference is because knockout animals undergo surgery in absence of the σ1R. Thus, pMCAO might promote increased excitotoxicity in σ1R-deficient mice via the direct and GPCR/HINT1-mediated activation of NMDAR signalling. In fact, NMDARs show increased responses to NMDA, indicating that σ1R dampens the activity of these glutamate receptors (24). Furthermore, the negative influence of cannabinoid type 1 receptors on NMDARs is lost in σ1R^−/−^ mice [21, 24, 39]. The protective effects of icv administration (3 nmol/mouse) of the sigma ligands S1RA, BD1047 and BD1063 were absent in mice with genetic σ1R deletion. These observations clearly indicate that σ1R is involved in post-injury ischaemic alterations, and sigma ligands partially modulate the neural injury that the stroke produces.

Following stroke, the disruption of the BBB is a crucial event in the secondary injury cascade, potentiating brain injury through a number of mechanisms, including cerebral oedema, increased neuroinflammatory response and haemorrhagic transformation [40, 41]. MMPs have been implicated in disease states that involve BBB dysregulation, tissue injury and cell death [42]. In particular, the expression and activity of MMP-9 have been found to increase in the ischaemic brain [7]. Accordingly, both inhibitors of MMPs and genetic deletion of MMP-9 attenuate BBB disruption and brain tissue infarction following ischaemia [43, 44]. Furthermore, clinical studies of stroke have found a correlation between MMP-9 levels in the blood and the rate of haemorrhagic transformation [45]. Notably, in this study, S1RA reduced oedema and decreased the MMP-9 activation associated with stroke. Moreover, this σ1R antagonist reduced the expression of inflammatory mediators such as nNOS that contribute to post-injury ischaemic conditions. Secondary damage can be caused by inflammation within the brain following cerebrovascular ischaemia and stroke. The precise causes of this inflammatory response have yet to be fully identified, but it is believed that necrotic cells and debris and reactive oxygen species are contributing factors. Within 24 h of ischaemic injury, these inflammatory triggers can activate microglia, which release an array of inflammatory and cytotoxic mediators that contribute to cell damage and death [46]. Accordingly, activated microglia have been identified in the cerebral cortex of animals exposed to transient focal cerebral ischaemia [47]. Microglial activation induced by cerebral ischaemia is concomitant with astrogliosis (the activation of astrocytes). During this reactive astrogliosis process, astroglial cells increase in size and number and undergo rapid expression of GFAP [47]. The degree of gliosis experienced following brain injury is thought to influence both structural and functional recovery such that certain levels are beneficial to the recovery process but excessive levels may be detrimental due to the associated neuroinflammatory responses [48–50]. Our data indicate that the administration of S1RA reduced reactive astrogliosis after ischaemic injury as demonstrated by decreases in microglial and astroglial proliferation and reduced expression of inflammatory markers. These observations were accompanied with demonstrable reductions in tissue injury and improved behavioural outcomes, indicating clear neuroprotective effects.

After stroke, the secondary overactivation of NMDARs contributes to brain damage [12, 51, 52]. Our model confirmed this finding via the increased phosphorylation of CaMKII observed after pMCAO; furthermore, the administration of S1RA decreased the NMDAR activation associated with stroke. In fact, σ1R forms a functional complex with the HINT1 protein at a molecular level to coordinate the activity of certain GPCRs with those of NMDARs [16]. In this scenario, the activation of these GPCRs leads to the PKC/Src-mediated phosphorylation of NMDARs, including their subsequent separation and activation [23, 53]. The antagonists of σ1Rs provoke the transfer of HINT1 proteins from activated GPCRs to NMDARs, thereby disrupting the GPCR-mediated activation of NMDARs. In our study, σ1R antagonists most likely prevented focal-activated GPCRs from enhancing the function of NMDARs in the surrounding area, thereby diminishing the average ischaemic damage. However, σ1R agonists can also reduce the effect of direct NMDAR activators. In fact, σ1R agonists, in the absence of GPCR activation, can promote GPCR–NMDAR association, and this complex diminishes the activity of the associated NMDARs. Thus, σ1R agonists that promote the association of inactive NMDARs with silent GPCRs reduce the pool of NMDARs that are susceptible to glycine/glutamate activation.

To the best of our knowledge, our study is the first to implicate σ1Rs in the neuronal damage promoted by experimental stroke. We report the beneficial effects of the selective σ1R antagonist S1RA on stroke-induced clinically relevant gait impairments. The use of drugs to target σ1R is an attractive therapeutic protection strategy compared with NMDAR antagonists that have a comparatively narrow therapeutic window.
